# Physical Exercise Reduces the Expression of RANTES and Its CCR5 Receptor in the Adipose Tissue of Obese Humans

**DOI:** 10.1155/2014/627150

**Published:** 2014-04-17

**Authors:** Engin Baturcam, Jehad Abubaker, Ali Tiss, Mohamed Abu-Farha, Abdelkrim Khadir, Fahad Al-Ghimlas, Irina Al-Khairi, Preethi Cherian, Naser Elkum, Maha Hammad, Jeena John, Sina Kavalakatt, Cynthia Lehe, Samia Warsame, Kazem Behbehani, Said Dermime, Mohammed Dehbi

**Affiliations:** ^1^Department of Biomedical Research, Dasman Diabetes Institute, 1180 Dasman, Kuwait; ^2^Queensland Children's Medical Research Institute, University of Queensland, Brisbane, QLD 4029, Australia; ^3^Fitness and Rehabilitation Centre, Dasman Diabetes Institute, 1180 Dasman, Kuwait; ^4^Department of Biostatistics and Epidemiology, Dasman Diabetes Institute, 1180 Dasman, Kuwait; ^5^King Fahad Specialist Hospital, Dammam 15215, Saudi Arabia; ^6^Diabetes Research Center, Qatar Biomedical Research Institute, Education City, P.O. Box 5825, Doha, Qatar

## Abstract

RANTES and its CCR5 receptor trigger inflammation and its progression to insulin resistance in obese. In the present study, we investigated for the first time the effect of physical exercise on the expression of RANTES and CCR5 in obese humans. Fifty-seven adult nondiabetic subjects (17 lean and 40 obese) were enrolled in a 3-month supervised physical exercise. RANTES and CCR5 expressions were measured in PBMCs and subcutaneous adipose tissue before and after exercise. Circulating plasma levels of RANTES were also investigated. There was a significant increase in RANTES and CCR5 expression in the subcutaneous adipose tissue of obese compared to lean. In PBMCs, however, while the levels of RANTES mRNA and protein were comparable between both groups, CCR5 mRNA was downregulated in obese subjects (*P* < 0.05). Physical exercise significantly reduced the expression of both RANTES and CCR5 (*P* < 0.05) in the adipose tissue of obese individuals with a concomitant decrease in the levels of the inflammatory markers TNF-**α**, IL-6, and P-JNK. Circulating RANTES correlated negatively with anti-inflammatory IL-1ra (*P* = 0.001) and positively with proinflammatory IP-10 and TBARS levels (*P* < 0.05). Therefore, physical exercise may provide an effective approach for combating the deleterious effects associated with obesity through RANTES signaling in the adipose tissue.

## 1. Introduction

Chronic low-grade inflammation and aberrant regulation of the stress response system are two prominent hallmarks of obesity, a major risk factor for the development of insulin resistance, type 2 diabetes, metabolic syndrome, and cardiovascular diseases [1, 2]. Sedentary lifestyles and excessive food intake are considered as key contributors to this chronic condition. The white adipose tissue has been identified as the predominant site of obesity-associated inflammatory reactions due to its infiltration by immune inflammatory cells such as monocytes, macrophages, Th1 T cells, and dendritic cells [2–4]. These immune cells, together with adipocytes and stromal vascular cells, constitute a cellular network that produces various inflammatory mediators. Obesity-induced inflammatory response impairs insulin signaling in insulin-responsive organs and causes systemic insulin resistance, which leads to a perturbation of glucose homeostasis and ultimately type-2 diabetes [5, 6]. Studies on mice indicated that obesity also alters the balance between pro- and anti-inflammatory activities in adipose tissue by promoting the phenotypic switch from M2 anti-inflammatory macrophages to M1 proinflammatory macrophages and thereby perpetuating further the inflammatory response and insulin resistance [5, 7].

Regulated upon Activation Normal T cells Expressed and Secreted (RANTES or CCL5) is a powerful proinflammatory mediator of the CC chemokine family that regulates the mobilization and, in certain cases, promotes survival of immune inflammatory cells from the bloodstream into tissues and other areas of injury and infection [3, 8, 9]. Although the chemotactic activity of RANTES on immune cells to injured and infected areas is beneficial, sustained production of RANTES is associated with several detrimental effects such as atherosclerosis [10, 11], arthritis rheumatoid [12], liver disease [13, 14], and viral infection [15] that share in common chronic inflammatory response. Consistent with its critical role in the pathophysiology of these chronic inflammatory-related diseases, treatments that interfere with RANTES signaling such as neutralizing antibody [16, 17], peptide mimetics [18, 19], and pharmacological inhibitors [20, 21] are associated with improved outcomes. RANTES orchestrates its effects through binding to one of its cognate receptors including CCR1, CCR3, and CCR5 [22, 23]. The crucial role of CCR5 in mediating the inflammatory response in adipose tissue was demonstrated recently by using CCR5 knockout mice that showed a dominant shift from M1-phenotype to M2-phenotype, which contributed to attenuated inflammatory response and improved impaired glucose tolerance and insulin sensitivity in response to diet-induced obesity [24].

Physical exercise is an important component of healthy lifestyle that is widely advocated as a first line for the treatment and management of obesity, insulin resistance, diabetes, and cardiovascular diseases [25–27]. Previous studies linked the protective effect of physical exercise to the improvement of the inflammatory and stress responses [28–30]. The mechanisms by which physical exercise mediates its anti-inflammatory effect are multiples and they include a reduction of visceral fat mass with subsequent decrease in the production of proinflammatory adipokines and a reduction in the circulating number of proinflammatory monocytes in favor of an increase in the circulating number of regulatory T cells [31, 32]. Other studies revealed also that physical exercise prevents monocytes and macrophages from infiltrating into adipose tissue and induces a phenotypic switching from M1-phenotype macrophages to M2-macrophages within the adipose tissue [33]. Other beneficial effects of physical exercise include attenuated activation of c-Jun NH_2_-terminal kinase [34] together with improvement of lipid, glucose, and oxidative homeostasis [35–37].

In the current study, we investigated the effect of a 3-month physical exercise on the expression and release of RANTES in obese subjects. Given the critical role of RANTES and its CCR5 receptor in promoting obesity-induced metabolic inflammation, we hypothesized that physical exercise may mediates its anti-inflammatory effect in part, by impairing RANTES signaling pathway via downregulation of RANTES and/or its CCR5 receptor. We report here for the first time a decrease in the expression of both RANTES and CCR5 receptor by physical exercise in the adipose tissue of obese humans.

## 2. Materials and Methods

### 2.1. Study Population

The study was conducted on adult male and female nondiabetic subjects consisting of 17 lean (BMI = 20–24.9 kg/m^2^) and 40 obese (BMI = 30–40 kg/m^2^). All subjects gave written informed consent and the study was approved by the Ethical Review Board of Dasman Diabetes Institute (Reference number:* RA-2010-003*).

Participants that followed any physical exercise within the last 6 months prior to this study as well as those with prior major illness or intake of medications or supplements known to influence the body composition or bone mass were excluded from the study. None of the participants that were enrolled in this study reported any cerebrovascular complication, kidney disease, asthma, and food and drug allergies and chronic viral infection. According to the medical questionnaire, none of the participants was under low-calorie or special diet. Furthermore, no specific diet or nutritional change was prescribed to our subject during the 3 months of physical exercise protocol as our aim was mainly to investigate the sole effect of physical exercise on those subjects.

### 2.2. Exercise Protocol

All eligible subjects were enrolled to a supervised exercise program at the Fitness and Rehabilitation Center (FRC) of Dasman Diabetes Institute. Prior to exercise prescription, each individual underwent a symptom-limited maximal incremental cardiopulmonary exercise test as well as a muscle strength and endurance test. The details of these tests and the exercise protocol have been previously described [38]. It consisted of a combination of both moderate intensity of aerobic exercise and resistance training using either treadmill or cycling. Each exercise session included 10 minutes of warming-up and cooling down steps at 50–60% of max HR, along with 40 minutes of the prescribed exercise program at 65–80% of max HR. For the duration of the 3-month period, participants exercised 3 to 5 times per week and they were instructed to reach and maintain the recommended heart rate range. This was achieved by regular monitoring of the heart rate during the aerobic training. Strength training was performed 2 to 3 times a week according to the program plan. Exercise intensity, duration, and blood pressures were recorded for each session. All trainings were supervised by qualified fitness professionals from FRC. To assess the effectiveness of the exercise, the same physical stress and fitness tests were performed for all subjects at the end of the exercise program.

### 2.3. Blood and Tissue Sampling

Venous peripheral blood and subcutaneous adipose tissue biopsies were obtained prior to the start of the 3-month protocol and within 2 to 3 days after its completion. PBMCs were prepared from blood using Ficoll-Hypaque density gradient centrifugation method. Plasma and serum were prepared using vacutainer tubes and then aliquoted and stored at −80°C. Subcutaneous superficial adipose tissue biopsies (~0.5 g) were obtained from the periumbilical area by surgical biopsy after a local anesthesia. Once removed, the biopsy was rinsed in cold PBS, divided into 4 pieces, and stored appropriately until assayed.

### 2.4. Anthropometric Measurements and Blood Biochemistry

Anthropometric measurements were taken at the baseline and after 3 months of exercise. Glucose and lipid profiles were measured on the Siemens Dimension RXL chemistry analyzer (Diamond Diagnostics, Holliston, MA). Hemoglobin A1_c_ (HBA1c) was determined using the Variant device (BioRad, Hercules, CA). Plasma levels of inflammatory and metabolic markers were measured using the Bio-plex 27-plex human cytokine and 12 Bio-plex human diabetes kits, respectively (BioRad, Hercules, CA). Data were analyzed with Bio-plex Manager software version 6 (BioRad, Hercules, CA). Lipid peroxidation was assessed by measuring plasma levels of malonaldehyde, using TBARs Assay Kit (Cayman Chemical Company, Ann Arbor, MI). Serum levels of ROS were determined using the OxiSelect ROS Assay Kit (Cell Biolabs Inc, San Diego, CA). All the above assays were carried out according to the instructions of the manufacturers.

### 2.5. Quantitative Real-Time PCR (qRT-PCR)

Total RNA was extracted from frozen adipose tissue and PBMCs using RNeasy Lipid Tissue Mini Kit and AllPrep RNA/Protein Kit, respectively (Qiagen, Inc., Valencia, CA). The cDNA was synthesized from total RNA sample using High Capacity cDNA Reverse Transcription Kits (Applied Biosystems, Foster City, CA). qRT-PCR was performed on Rotor-Disc 100 system using SYBR Green normalized to* Gapdh* (Qiagen, Inc., Valencia, CA). PCR primer used were as follows: RANTES For., 5′-TTTGCCTACATTGCCCGC-3′; RANTES Rev., 5′-TTTCGGGTGACAAAGACGACT-3′; CCR5 For., 5′-CAAAAAGAAGGTCTTCATTACACC-3′ and* Ccr5* Rev., 5′-CCTGTGCCTCTTCTTCTCATTTCG-3′; TNF-*α* For., 5′-AGAGGGAAGAGTTCCCCAGG-3′; TNF-*α* Rev., 5′-ATTGGCCAGGAGGGCATT-3′;* IL-6* For., 5′-AGAAAGGAGAGTCACAGGTGAGC-3′;* IL-6* Rev., 5′-TGTCTGGGAAAGAATACCAGAA-3′;* Gapdh* For., 5′-AACTTTGGCATTGTGGAAGG-3′; and* Gapdh* Rev., 5′-TGTGAGGGAGATGCTCAGTG-3′. Relative expression was assessed by using the ΔΔCT method [39].

### 2.6. Western Blot Analysis

Cell lysates were prepared from PBMCs by the addition of RIPA buffer (50 mM Tris-HCl pH 7.5, 150 mM NaCl, 1% Triton x100, 1 mM EDTA, 0.5% Sodium deoxycholate, and 0.1% SDS). Protein concentration was determined by Bradford method using globulin as a standard and 20 *μ*g of proteins was resolved on 10% SDS-PAGE gels. Proteins were transferred onto PVDF membranes and probed with primary and secondary antibodies using standard protocols. Protein bands were visualized by chemiluminescence and the images were captured by using the Versadoc 5000 system (BioRad, Hercules, CA). The primary antibodies used in this study are raised against RANTES (Abcam, Cambridge, MA) and Actin (Santa Cruz Biotechnology, Santa Cruz, CA). For densitometric analysis, the intensity of the bands was determined using Quantity One Software (BioRad, Hercules, CA).

### 2.7. Immunohistochemistry

Formalin-fixed and paraffin-embedded adipose tissue sections were deparaffinized and rehydrated prior to antigen retrieval by boiling in the unmasking solution (Dako, Denmark). The endogenous peroxidase was quenched using 3% H_2_O_2_ for 60 min at RT. Sections were blocked with 5% fat-free milk for 60 min at RT followed by 1% BSA for another 60 min and then incubated at 4°C for overnight with primary antibodies against RANTES (Abcam, Cambridge, MA), TNF-a (Abcam, Cambridge, MA), IL-6 (Novus Biologicals, Littleton, CO), and Phospho-JNK (Cell Signaling Technology, Inc., Danvers, MA). Staining with horseradish conjugated secondary antibody (Dako, Denmark) was done for 60 minutes at RT. Colors were developed using DAB kit (Dako, Denmark) and sections were counterstained with hematoxylin (Sigma Aldrich, St. Louis, MO). Quantification of the immunohistochemical data was done using Aperio software version 6.3 (Molecular Devices, Downingtown, PA) with an established arbitrary threshold.

### 2.8. Flow Cytometry Analysis

Approximately 5 × 10^5^ of frozen PBMCs were thawed and washed with RPMI medium supplemented with 10% FBS, antibiotics, and L-glutamine (complete media). For RANTES staining, cells were cultured overnight in complete media at 37°C and then washed with PBS containing 2% FBS followed by FACS buffer (BD Biosciences, San Jose, CA) and then costained with anti-human APC-conjugated RANTES (R&D Systems), FITC-conjugated CD3 (MCA463F, AbD Serotec, Raleigh, NC), and PE-conjugated CD14 (FAB3832P, R&D Systems, Minneapolis, MN) antibodies for 45 minutes on ice. Cells were then washed twice with permeabilization buffer (eBioscience, San Diego, CA) and resuspended in 300 *μ*L FACS buffer for analysis. Regarding CCR5 staining, cells were allowed to recover for 1-2 hours in complete media. The cells were then cultured in complete media in presence of 2 *μ*M monensin (eBioscience, San Diego, CA) at 37°C and 5% CO_2_ for 16 hours. PBMCs were then washed with FB and surface stained simultaneously with FITC-conjugated CD3 (MCA463F) and PE-conjugated CD14 (FAB3832P) antibodies. After 45 minutes of incubation on ice in the dark, cells were washed twice and fixed with fixation buffer (eBioscience, San Diego, CA) for 20 minutes. Cells were then washed twice with permeabilization buffer (eBioscience, San Diego, CA) according to manufacturer's recommendation followed by staining with CCR5-APC antibody (FAB1802A, R&D Systems, Minneapolis, MN) for 20 minutes in the dark. Finally, the cells were resuspended in 300 *μ*L FACS Buffer for analysis. Controls for specific labeling were prepared with isotype-matched controls for each sample. Acquisition and analysis were carried out on a FACS Canto II flow cytometer using FACSDiva software (BD Biosciences, San Jose, CA). Typically, 30,000 events for PBMCS were acquired. After forward scatter (FSC) and side scatter (SSC) gating on either lymphocytes or monocytes the RANTES expression level was determined in the CD3 as well as in CD14 subsets.

### 2.9. Statistical Analysis

Statistical analyses were performed with SAS version 9.2 (SAS Institute Inc, Cary, NC). Unless otherwise stated, all descriptive statistics for the variables in the study were reported as means ± standard error. Nonparametric Mann-Whitney test was used to determine significance of difference in means between the two groups as indicated in the figure legends. Correlations between variables were calculated with the Spearman's rank correlation test. Differences were considered statistically significant at *P* values less than 0.05.

## 3. Results

### 3.1. Baseline Characteristics of Study Population

The physical characteristics of the two groups, namely, lean (*n* = 17) and obese (*n* = 40), enrolled in this study are displayed in Table 1. As expected, body mass index, percent body fat, and waist and hip circumferences were significantly higher in obese group compared to lean group (*P* < 0.0001). Obese subjects had also higher systolic blood pressure and higher levels of triglycerides (*P* < 0.05); however, there was no significant difference in the cardiopulmonary parameters between the two groups. Obese subjects had higher levels of glucose and HbA1c (*P* < 0.05) in spite of not being diabetic. Compared to lean group, the metabolic and inflammatory profiles are dysregulated in obese group as indicated by increased levels of leptin (*P* = 0.0006), resistin (*P* = 0.04), IP-10 (*P* = 0.0008), and RANTES chemokines (*P* = 0.023). No significant difference was observed in the other metabolic and inflammatory mediators as well as the oxidative stress markers (Table 1 and data not shown).

### 3.2. Levels of RANTES Protein and mRNA Are Increased in the Adipose Tissue but Not in PBMCs of Obese Subjects

**Figure 1:**
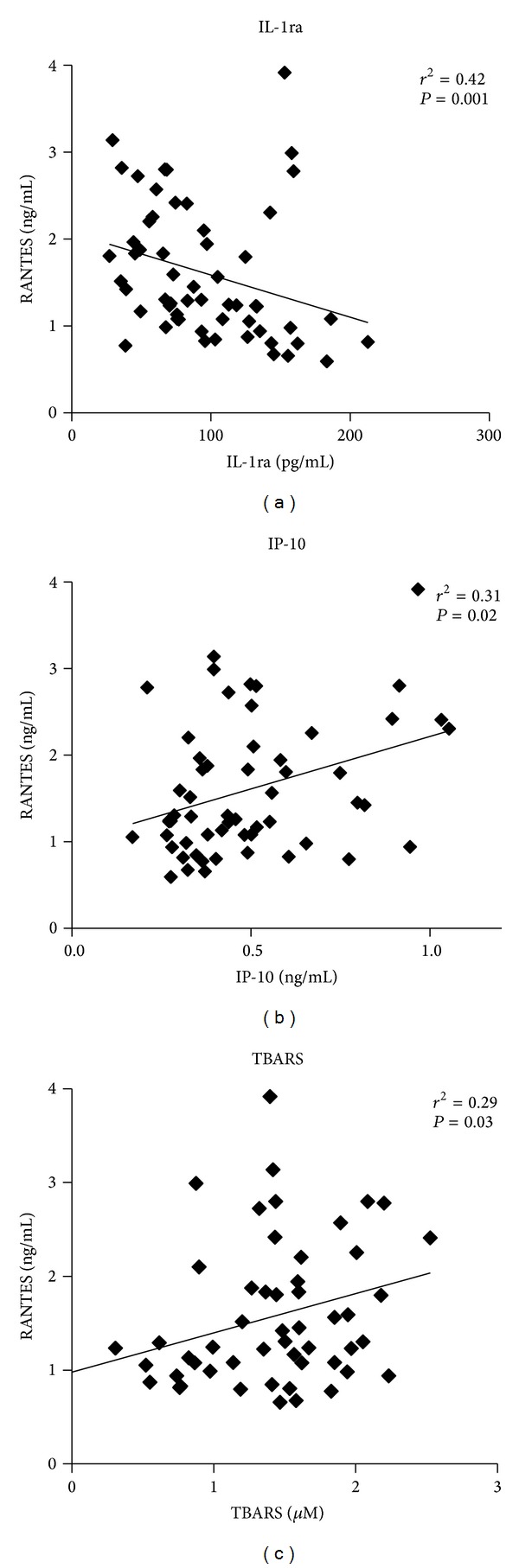
Correlation analysis of circulating RANTES with key inflammatory makers. In a mixed population of lean (*n* = 17) and obese (*n* = 40), circulating RANTES correlated negatively with the IL-1ra levels and positively with IP-10 chemokine levels and TBARS activity. Correlations were assessed using Spearman's rank correlation coefficient.

**Figure 2:**
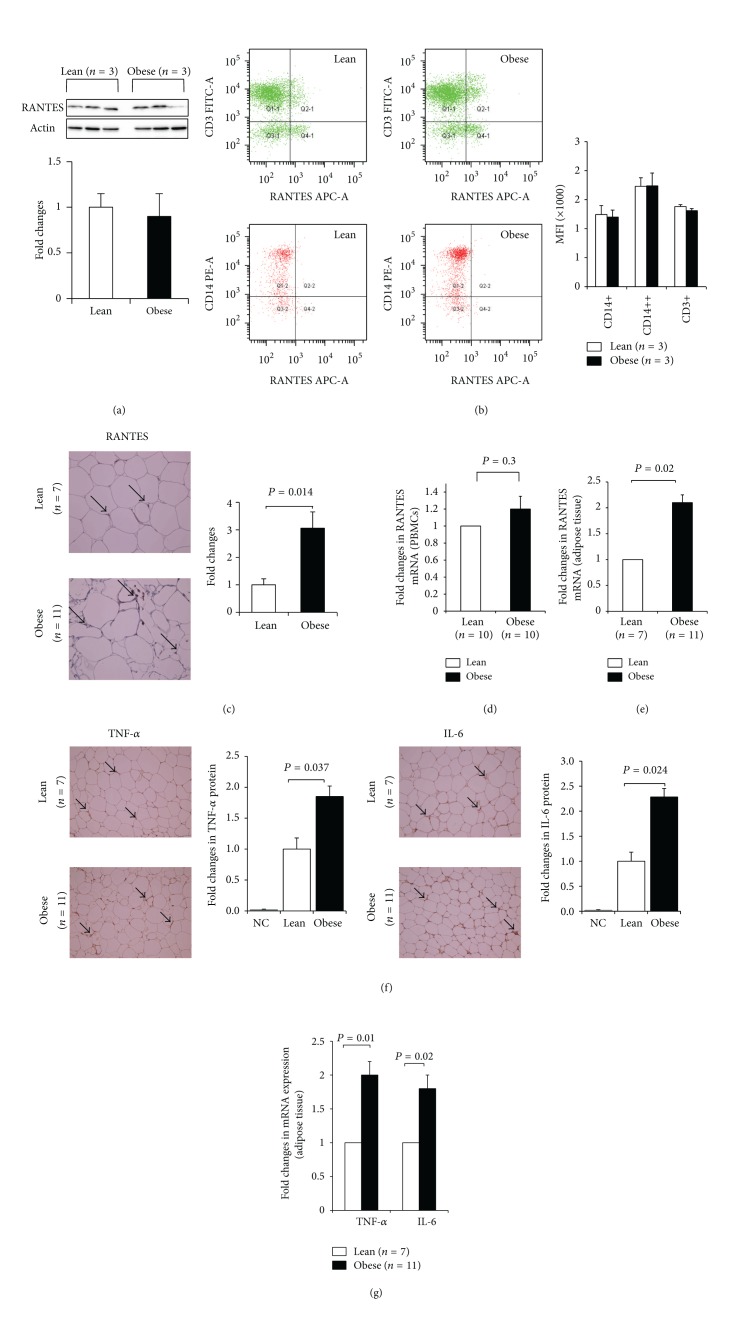
Expression of RANTES in obese subjects. (a) Western blot analysis of RANTES expression in PBMCs from lean and obese subjects. The bands reacting with anti-RANTES antibody were quantified as described in Section 2 and the relative intensity was determined after correction with Actin that was used as internal control to monitor loading efficiency. The blots shown are representative of three independent experiments with consistent results. The data are presented in the form of a bar graph on the right of the figure as fold changes of RANTES protein expression in obese compared to lean subjects. (b) Characterization of the monocyte subpopulations and T cells in peripheral blood from lean and obese participants. Monocytes subsets were defined by staining for CD14 (PE), T cells by CD3 (FITC), and expression of intracellular RANTES (APC) was analyzed. Left and right upper panels are representative dot plots of CD3 and RANTES expression on/in T cells from lean and obese participants, respectively. Left and right lowest panels are representative dot plots of CD14 and RANTES expression on/in monocyte subsets from lean and obese participants, respectively. The double-positive populations (i.e., CD3+RANTES+, CD14+RANTES+, and CD14++RANTES+) were analyzed for mean RANTES fluorescence intensity. (c) Analysis of RANTES expression by immunohistochemistry (IHC) in the subcutaneous adipose tissues from lean and obese nondiabetic participants. Aperio software was used to quantify positive staining (indicated by arrows) and quantified values relative to lean controls are plotted in a bar graph at the bottom. (d and e) Analysis of RANTES mRNA expression by quantitative real-time PCR (qRT-PCR) between lean and obese subjects. Total RNA was isolated from PBMCs (d) and adipose tissue biopsies (e). The data are presented as fold changes in obese compared to lean subjects after normalization with the GAPDH reference gene. (f and g) Analysis of TNF-*α* and IL-6 expression at the protein level by IHC (f) and at the mRNA level by qRT-PCR (g) in the adipose tissue from lean and obese subjects. In IHC experiments, Aperio software was used to quantify positive staining as indicated above and the values are illustrated at the bottom as fold changes compared to lean. Mann-Whitney test was used to determine significance of difference between the lean and obese subjects. For each experiment, the sample size from each group is indicated by *n*.

In agreement with our recent investigation [38], obese subjects had higher levels of RANTES in the circulation that correlated negatively with IL-1ra (*r* ^2^ = 0.42; *P* = 0.001) and positively with IP-10 (*r* ^2^ = 0.31; *P* = 0.02) and TBARS levels (*r* ^2^ = 0.29; *P* = 0.03) (Figure 1(a)). To investigate the endogenous expression of RANTES between the two groups at the protein level, we determined its expression in PBMCs and adipose tissue by western blot, flow cytometry, and immunohistochemistry (IHC). As shown in Figure 2(a), there was no difference in the expression of RANTES protein in PBMCs between lean and obese subjects (*n* = 3 for each group). Consistent with this finding, flow cytometry indicated that RANTES levels were comparable between lean and obese subjects (*n* = 3 for each group) in both monocytes and lymphocytes (Figure 2(b)). By contrast to PBMCs, the expression of RANTES protein in the adipose tissue as monitored by IHC studies was significantly higher in obese (*n* = 11) compared to lean (*n* = 7) subjects (*P* = 0.01; Figure 2(c)). The differential expression of RANTES between PBMCs and adipose tissue in lean and obese subjects was also confirmed at the mRNA level by using quantitative real-time PCR (qRT-PCR) and the results are displayed in Figures 2(d) and 2(e). Accordingly, there was no change in RANTES mRNA levels in PBMCs from lean and obese subjects (*n* = 10 for both lean and obese, Figure 2(d)), whereas the levels of RANTES mRNA in the adipose tissue were significantly higher in obese (*n* = 11) compared to lean (*n* = 7) subjects (*P* = 0.02, Figure 2(e)). Under the same conditions, the endogenous expression of the two key inflammatory markers TNF-*α* and IL-6 in the adipose tissue were significantly increased in obese subjects both at the protein level (*P* < 0.05, Figure 2(f)) and mRNA level (*P* < 0.05, Figure 2(g)).

### 3.3. CCR5 mRNA Is Upregulated in the Adipose Tissue of Obese Subjects

**Figure 3:**
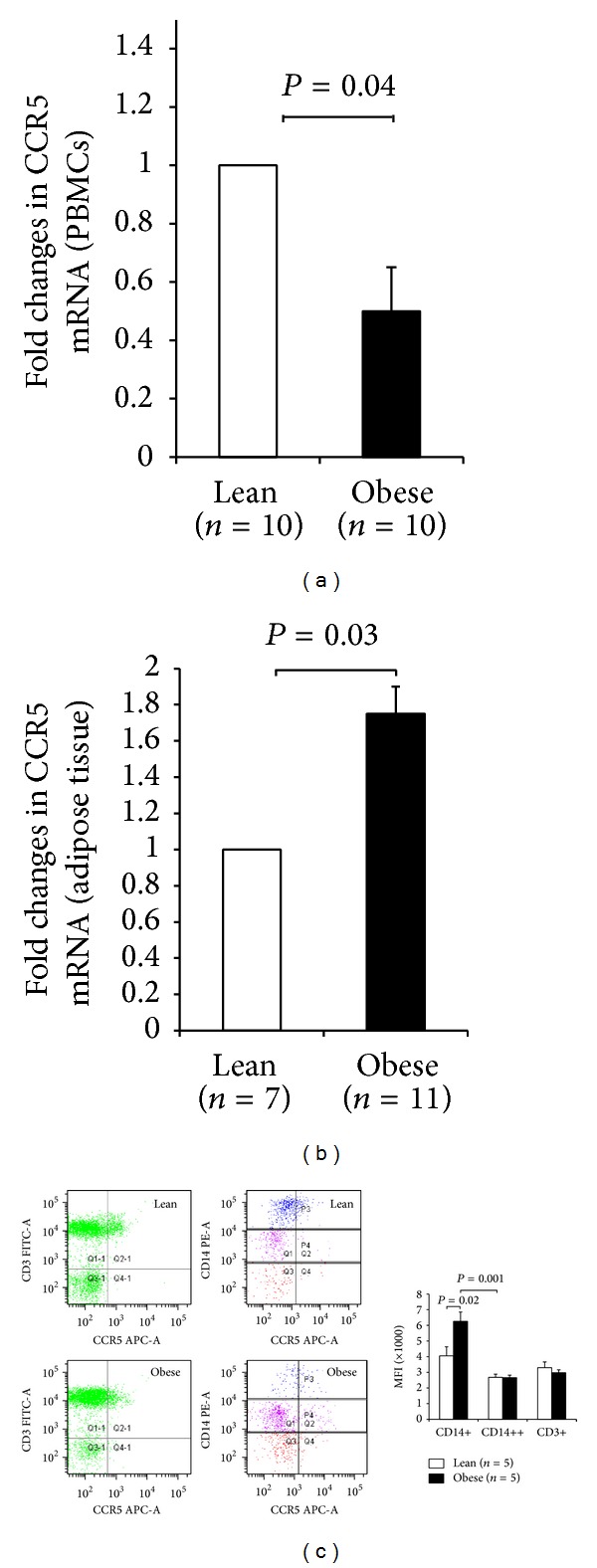
Differential regulation of CCR5 in PBMCs and adipose tissue is associated with obesity. CCR5 gene expression was measured by qRT-PCR in PBMCs (a) from lean and obese nondiabetic subjects and in the adipose tissue (b) from lean and obese nondiabetic subjects. The data are presented as fold changes in obese compared to lean subjects after normalization with the GAPDH reference gene. (c) Characterization of the monocyte subpopulations and T cells in peripheral blood from lean and obese subjects. Monocytes subsets were defined by staining for CD14 (PE), T cells by CD3 (FITC), and expression of CCR5 (APC) and then were analyzed. Gates P4 and P3 define the CD14+ and CD14++ subsets, respectively. Left and right upper panels are representative dot plots of CD3 and CCR5 expression on T cells from lean and obese subjects, respectively. Left and right lower panels are representative dot plots of CD14 and RANTES expression on monocyte subsets from lean and obese participants, respectively. The double-positive populations (i.e., CD3+CCR5+, CD14+CCR5+, and CD14++CCR5+) were analyzed for mean CCR5 fluorescence intensity. For each experiment, the sample size from each group is indicated by *n*.

We next examined the expression profile of CCR5 receptor between lean and obese subjects using PBMCs and adipose tissue. Data shown in Figure 3 indicated a peculiar expression pattern of CCR5 receptor. Indeed, there was a clear upregulation of* Ccr5* mRNA in the adipose tissue of obese (*n* = 11) compared to lean (*n* = 7) subjects (*P* = 0.03, Figure 3(b)). By contrast,* Ccr5* mRNA was significantly downregulated in PBMCs of obese subjects compared to lean subjects (*n* = 10 for both lean and obese) (*P* = 0.04, Figure 3(a)). On the other hand, flow cytometry analysis carried out on PBMCs from lean and obese (*n* = 5 for each group) revealed a significant and unique increase of CCR5 in CD14+ monocyte subset from obese subjects (*P* = 0.02, Figure 3(c)). The specific mean fluorescence intensity for CCR5 in obese individuals revealed a higher signal for CD14+ monocytes compared to CD14++ monocytes (*P* = 0.001, Figure 3(c)). Lean controls displayed the same trend but to a lesser extent.

### 3.4. Effect of Physical Exercise on RANTES and CCR5 Expression

**Figure 4:**
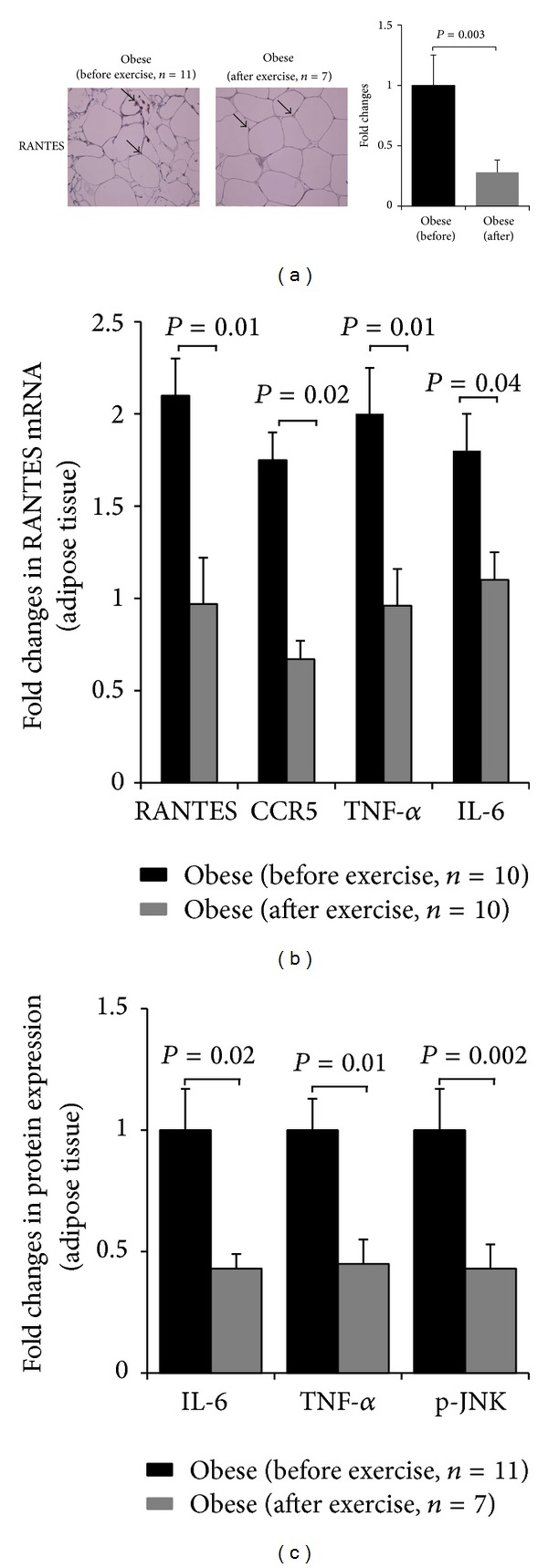
Physical exercise reduces the expression of RANTES and CCR5 in the adipose tissue. (a) IHC staining with RANTES antibody using subcutaneous adipose tissues from obese subjects before and after 3 months of physical exercise. Aperio software was used to quantify positive staining in obese before and after physical exercise. The data are plotted in a bar graph on the right of the figure as fold changes of RANTES protein expression in obese subjects before and after the physical exercise program. (b) qRT-PCR analysis of RANTES, CCR5, TNF-*α*, and IL-6 mRNA expression in the adipose tissue from obese before and after 3 months of physical exercise. (c) Graphic representation of IHC staining with TNF-*α*, anti-IL-6, and anti-Phospho-JNK antibodies using subcutaneous adipose tissues from obese subjects before and after 3 months of physical exercise. Aperio software was used to quantify positive staining in obese before and after physical exercise. Paired* t*-test for two group analysis was done to compare the expression of proteins and mRNA in obese before and after exercise. For each experiment, the sample size from each group is indicated by *n*.

We previously reported the effectiveness of our physical exercise protocol on improving the physical, clinical, and metabolic parameters on obese subjects [38]. Accordingly, there was a significant reduction of PBF and SBP and increase in *V*_O_2_*Max*⁡_ along with a decrease in TBARS levels and a reduction of inflammatory markers TNF-*α* and IL-6 in the circulation [38]. However, physical exercise did not reduce the levels of RANTES in the circulation [38]. To investigate whether physical exercise has an impact on the endogenous expression of RANTES and CCR5, qRT-PCR and IHC were carried out on adipose tissue from obese subjects before and after the exercise program. As shown in Figure 4(a), IHC carried out on adipose tissue from obese subjects before (*n* = 11) and after (*n* = 7) the exercise program indicated a significant decrease in the expression of RANTES by physical exercise (*P* = 0.003). qRT-PCR performed on obese before and after the exercise program (*n* = 10 for each group) confirmed the reduction of RANTES mRNA expression by physical exercise (*P* = 0.01, Figure 4(b)). Likewise, CCR5 mRNA was significantly reduced by physical exercise in the adipose tissue (*P* = 0.02, Figure 4(b)). Using the same samples, we observed a decrease in the endogenous expression of TNF-*α* and IL-6 both at the mRNA level (*P* < 0.05, Figure 4(b)) and protein level (*P* < 0.05, Figure 4(c)).

Since obesity is known to induce the phosphorylation of JNK protein, we measured the levels of phosphorylated JNK in adipose tissue by IHC before (*n* = 11) and after (*n* = 7) the physical exercise program. Data shown on Figure 4(c) indicated that physical exercise decreases significantly the levels of phosphorylated JNK (*P* = 0.002) in obese in a manner that was concomitant with a decrease of CCR5 and its ligand RANTES. No effect was observed for total JNK before and after exercise in obese subjects (data not shown). Taken together, these data suggest that exercise is interfering with obesity-mediated expression of RANTES and CCR5.

## 4. Discussion

Chronic low-grade inflammation state is a key feature of obesity and it is caused mainly by infiltration of macrophages and other inflammatory cells into the adipose tissue. The emerging role of RANTES signaling pathway to this chronic condition is well established. The present study explored the effect of physical exercise on the expression of RANTES and its main receptor* Ccr5* in obese humans. Our focus on CCR5 receptor was based on a recent study in which CCR5 knockout mice were protected from obesity-induced adipose tissue inflammation and insulin resistance [24]. In addition, it has been previously shown that obesity triggers a modest change in the expression of CCR3 and no change in that of CCR1 [17]. The main findings of our current investigation are as follows: (1) the expression of both RANTES and CCR5 was significantly higher in the subcutaneous adipose tissue of obese subjects, (2) by contrast to the adipose tissue,* Ccr5* was downregulated in PBMCs of obese subjects compared to lean subjects but there was no significant difference in the expression of RANTES between the two groups, and (3) physical exercise corrected the dysregulated expression of both RANTES and CCR5 in the subcutaneous adipose tissue but has a marginal effect on circulating levels of RANTES. While our findings showing increased expression of RANTES and CCR5 in the adipose tissue are consistent with previous clinical and animal studies [17, 40], the downregulation of their expression by physical exercise is novel. Based on the crucial role of RANTES/CCR5 signaling pathway in the pathology of obesity and its associated complications, our findings add further evidence that physical exercise might be one of nonpharmacologic approaches that can attenuate RANTES/CCR5 signaling pathway and thereby mitigating the inflammatory and metabolic stress triggered by obesity.

RANTES is a powerful proinflammatory chemokine that controls the trafficking of immune inflammatory cells such as monocytes, macrophages, Th1 T cells, and dendritic cells from the circulation into various tissues including adipose tissue [3, 9]. Previous studies carried out on obese humans and rodents reported high levels of RANTES as well as increased frequency of macrophages in the adipose tissue that were concomitant with increased inflammatory response, insulin resistance, and type 2 diabetes [5, 17, 41–44]. Our findings showing high levels of RANTES in the adipose tissue of obese subjects at both mRNA and protein levels corroborate these pioneer studies that associated RANTES with obesity. Unlike the adipose tissue, the levels of RANTES were however comparable between lean and obese subjects as monitored by western blotting, flow cytometry, and qRT-PCR. The mechanism underlying this differential expression of RANTES between PBMCs and adipose tissue remains to be investigated but there is evidence for depot-specific differences that dictate the levels of RANTES released by various adipose tissues in humans [44].

One of the key characteristics of macrophages that infiltrate the adipose tissue is the heterogeneity of their phenotype and depending on their polarization status; they can be proinflammatory macrophages (M1-phenotype) secreting various inflammatory mediators such as TNF-*α*, IL-6, and iNOS [45, 46] or anti-inflammatory macrophages (M2-phenotype) that secrete anti-inflammatory cytokines such as IL-10 [41, 47]. In obesity, the differentiation of M2 into M1 macrophages is considered as a major event that sustains chronic inflammation [5]. Indeed, studies on mice indicated that obesity induces a shift in macrophage balance towards the M1-phenotype macrophages that perpetuate further the inflammatory response and insulin resistance [5, 46]. In our study population, we did not evaluate the status of M1- and M2-phenotypes, which may represent a limitation to this investigation. However, in our study the high levels of circulating RANTES correlated negatively with the anti-inflammatory IL-1ra and positively with the levels of proinflammatory IP-10 chemokine and TBARS supporting its role in mediating inflammation.

In the current study, we have also investigated the expression pattern of CCR5 in obese humans and similar to our findings with RANTES, both mRNA and protein levels of* Ccr5* were increased in the adipose tissue of obese compared to lean group. The observed increase is consistent with recent studies both on humans and rodents [24, 40]. The direct role of CCR5 in the regulation of obesity-induced adipose tissue inflammation and development of insulin resistance was recently demonstrated using CCR5 knockout mice [24]. A dominant shift occurred from proinflammatory M1 to anti-inflammatory M2 macrophage phenotypes, which contributed to an improved impaired glucose tolerance and insulin sensitivity in response to diet-induced obesity in these animals [24].

Unexpectedly, our data demonstrated that, unlike the adipose tissue, the expression of* Ccr5* mRNA was significantly downregulated in PBMCs from obese individuals. To the best of our knowledge, our data is the first to report the downregulation of* Ccr5* mRNA in the PBMCs of obese individuals. This observed downregulation is intriguing and might suggest a complex regulation process of its transcription. The altered expression of CCR5 in PBMCs has been described in rheumatoid arthritis [48] and in women with systemic lupus erythematosus [49]. It is possible that other receptors for RANTES such as CCR1 and CCR3 may be compensating for the reduced expression of CCR5 in obese subjects. Interestingly, CCR5 expression on CD14+ monocyte subset appears to be upregulation in comparison with CD14++ subset in obese individuals. CD14+ cells are known to exhibit a macrophage-like phenotype with enhanced antigen-presenting capacities, higher endothelial affinity, and they are potent producers of proinflammatory cytokines [50]; thus, they may contribute to the development of chronic inflammation.

Of particular interest, our study emphasized more on the role of physical exercise in regulating the expression of RANTES and its receptor CCR5. It is well established that exercise reduces the risk of chronic metabolic and cardiorespiratory diseases by modulating the inflammatory and stress responses [51, 52]. Our data showed for the first time that physical exercise significantly reduced the expression of both RANTES and* Ccr5* receptor in the adipose tissue in a manner that was concomitant with decreased PBF and improved cardiopulmonary performance as monitored by decreased SBP and increased *V*_O_2_*Max*⁡_. The reduced expression of RANTES and CCR5 by physical exercise was also consistent with attenuated inflammatory response as indicated by decreased TNF-*α* and IL-6 in the circulation and reduced levels of TBARS. Our finding revealed also that exercise-mediated decrease in the expression of RANTES and* Ccr5* in the adipose tissue was not translated to a decrease in the circulating levels of RANTES. The lack of parallel changes between systemic and endogenous levels of RANTES after exercise raises the question regarding the role of RANTES in the circulation. Earlier study indicated that systemic levels of RANTES are 100-fold higher than those being released from any of the adipose tissue depots [44]. Therefore, it is sensible to suggest that while adipose tissue is able to produce this chemokine, its contribution to systemic levels may be less significant.

Physical exercise is also known to inhibit metabolic stress in part by dephosphorylating and thus inactivating JNK stress kinase [34, 53, 54]. In agreement with these findings, our data indicate that there was a significant increase in the levels of phosphorylated JNK along with high levels of RANTES and CCR5 in obese subjects that were all reduced by physical exercise (Figure 4(c)). Whether there is a direct crosstalk between RANTES/CCR5 signaling pathway and JNK signaling or not remains to be investigated, despite the fact that CCR5 knockout mice displayed attenuated signaling via NF-*κ*B and JNK [24].

In conclusion, the dysregulation of RANTES and its negative correlation with the metabolic and stress responses are supporting the pathological role of RANTES in obesity. Our results also provide strong evidence that physical exercise downregulates the expression of both RANTES and* Ccr5* in the adipose tissue. These findings give strong support to the concept that physical exercise can be used as a nonpharmacologic approach to mitigate the complications associated with obesity, particularly inflammation, in part by attenuating RANTES/CCR5 signaling in the adipose tissue.

## Figures and Tables

**Table 1:** Physical, clinical, and biochemical characteristics of the study population at baseline.

	Lean (*n* = 17)	Obese (*n* = 40)	*P* value
Physical and clinical characteristics			
Gender (M/F)	6/11	22/18	*0.18 *
Age (year)	37.24 ± 2.49	43.45 ± 1.87	*0.06 *
BMI (kg/m^2^)	22.78 ± 0.55	34.25 ± 0.48	*<0.0001 *
PBF (%)	25.86 ± 1.08	38.67 ± 0.73	*<0.0001 *
Waist (cm)	76.04 ± 5.46	107.53 ± 2.82	*<0.0001 *
Hip (cm)	84.08 ± 5.73	115.15 ± 2.68	*<0.0001 *
Resting HR (beat/min)	82.17 ± 7.89	78.65 ± 1.98	*0.94 *
SBP (mmHg)	115.33 ± 4.37	127.94 ± 3.12	*0.09 *
DBP (mmHg)	80.00 ± 2.58	81.59 ± 2.32	*0.94 *
*V*_O_2_ Max_ (mL/kg/min)	20.09 ± 1.18	18.12 ± 1.17	*0.22 *
Metabolic markers			
Cholesterol (mmol/L)	4.97 ± 0.23	5.32 ± 0.15	*0.20 *
HDL (mmol/L)	1.28 ± 0.06	1.14 ± 0.04	*0.06 *
LDL (mmol/L)	3.22 ± 0.22	3.35 ± 0.14	*0.62 *
TG (mmol/L)	1.03 ± 0.22	1.72 ± 0.14	*0.013 *
Glucose (mmol/L)	4.96 ± 0.18	5.43 ± 0.12	*0.035 *
HBA1c (%)	5.53 ± 0.10	5.85 ± 0.07	*0.013 *
C-peptide (ng/mL)	2.67 ± 0.29	3.03 ± 0.18	*0.29 *
Insulin (ng/mL)	2.59 ± 0.46	3.47 ± 0.29	*0.12 *
Leptin (ng/mL)	4.71 ± 0.90	8.66 ± 0.58	*0.0006 *
PAI-1 (ng/mL)	3.26 ± 0.38	3.64 ± 0.24	*0.41 *
Resistin (ng/mL)	1.22 ± 0.11	0.94 ± 0.07	*0.04 *
Visfatin (ng/mL)	9.78 ± 2.13	10.23 ± 1.38	*0.86 *
Inflammatory markers			
TNF-*α* (pg/mL)	25.64 ± 3.77	28.75 ± 2.13	*0.49 *
IL-1*β* (pg/mL)	1.25 ± 0.19	1.33 ± 0.12	*0.72 *
IL-1ra (pg/mL)	105 ± 11	92.7 ± 7.3	*0.38 *
IL-4 (pg/mL)	2.30 ± 0.26	2.09 ± 0.17	*0.50 *
IL-6 (pg/mL)	5.56 ± 0.56	4.99 ± 0.36	*0.40 *
IL-10 (pg/mL)	2.29 ± 0.71	2.45 ± 0.45	*0.85 *
IP-10 (pg/mL)	344 ± 50	558 ± 32	*0.0008 *
MCP-1 (pg/mL)	9.32 ± 1.00	10.03 ± 0.64	*0.56 *
MIP-1a (pg/mL)	9.26 ± 3.32	3.41 ± 1.80	*0.13 *
MIP-1b (pg/mL)	22.42 ± 6.94	30.19 ± 4.44	*0.36 *
RANTES (ng/mL)	1.24 ± 0.18	1.75 ± 0.12	*0.023 *
Oxidative stress markers			
ROS (mM)	1.42 ± 0.06	1.51 ± 0.06	*0.33 *
TBARS (*μ*M)	1.22 ± 0.12	1.49 ± 0.08	*0.07 *

Data were adjusted for age and gender and presented as mean ± SE. BMI: body mass index, PBF: percent body fat, HR: heart rate, SBP: systolic blood pressure, DBP: diastolic blood pressure, V_O_2_ Max_: maximum oxygen consumption, HDL: high density lipoprotein, LDL: low density lipoprotein, and TG: triglycerides.
